# Incidence of glaucomatous visual field loss after two decades of follow-up: the Rotterdam Study

**DOI:** 10.1007/s10654-017-0270-y

**Published:** 2017-06-12

**Authors:** Henriët Springelkamp, Roger C. Wolfs, Wishal D. Ramdas, Albert Hofman, Johannes R. Vingerling, Caroline C. Klaver, Nomdo M. Jansonius

**Affiliations:** 1 Department of Epidemiology, ErasmusMC, Rotterdam, The Netherlands; 2 Department of Ophthalmology, ErasmusMC, Rotterdam, The Netherlands; 3 Department of Ophthalmology, University of Groningen, University Medical Center Groningen, P.O. Box 30001, 9700 RB Groningen, The Netherlands

**Keywords:** Glaucoma, Incidence, Optic nerve, Visual field, Intraocular pressure

## Abstract

To determine the incidence of glaucomatous visual field loss (GVFL) two decades after the start of the Rotterdam Study, and to compare known risk factors for open-angle glaucoma (OAG) between different clinical manifestations of OAG. Of 6806 participants aged 55 years and older from the population-based Rotterdam Study, 3939 underwent visual field testing at baseline and at least one follow-up round. The ophthalmic examinations included optic disc assessment and measurements of intraocular pressure (IOP), refractive error, diastolic blood pressure (DBP), and height and weight. The incidence rate of GVFL was calculated. Associations with the risk factors age, gender, baseline IOP, family history, myopia, DBP, and body-mass index [BMI] were assessed using Cox regression, with different clinical manifestations of OAG as outcome measure (glaucomatous optic neuropathy (GON), GVFL, GVFL and GON, GVFL without GON, and GON without GVFL). Median follow-up was 11.1 (IQR 6.8–17.2; range 5.0–20.3) years. The incidence rate of GVFL was 2.9 (95% confidence interval 2.4–3.4) per 1000 person years (140 cases with incident GVFL in one (n = 113) or both (n = 27) eyes). Baseline IOP and age were significantly associated with all OAG outcomes (all *p* < 0.001); BMI showed a non-significant protective effect in all outcomes (*p* = 0.01 to *p* = 0.09). Gender, myopia, and DBP were not associated with any outcome. Our study provides an estimate of the long-term incidence of GVFL in a predominantly white population. The development of GVFL was strongly associated with baseline IOP and age. Risk factor profiles were similar for the different outcomes.

## Introduction

Glaucoma is a group of diseases that affect the optic nerve. Primary open-angle glaucoma (OAG) is one of the most common forms of glaucoma. It is characterized by loss of retinal ganglion cells (RGCs) and thinning of the retinal nerve fiber layer (RNFL). Another hallmark is excavation of the optic nerve head (ONH), glaucomatous optic neuropathy (GON). These structural changes are visible by fundoscopy or can be assessed with imaging techniques like scanning laser ophthalmoscopy, scanning laser polarimetry, or optical coherence tomography. In general, loss of RGCs and RNFL leads to visual field defects. This functional loss can be measured by perimetry. The surprisingly weak association between structural and functional changes in individual patients is one of the major unsolved issues in glaucoma.

In a general ophthalmology clinical setting, an examination of the ONH and a measurement of the intraocular pressure (IOP) belong to standard care, whereas perimetry does not. Perimetry will only be performed in patients with a suspicious appearance of the ONH or an elevated IOP. This biases the clinical manifestation of OAG towards high-tension glaucoma (HTG) and/or pronounced ONH abnormalities. The clinical impression that normal tension glaucoma (NTG) patients have more pronounced ONH abnormalities than HTG patients (at a given level of visual field loss) might be the result of this bias, since HTG can be detected after an IOP measurement in the absence of a suspicious ONH appearance. Population-based studies that perform perimetry in all subjects avoid this bias. This makes these studies unique for studying the different clinical manifestations of OAG, for example differences in the structure–function relationship between HTG and NTG. Interestingly, NTG with an apparently normal ONH appearance, the manifestation of OAG that is very difficult to detect in a clinical setting, is all but rare in a population-based setting [1, 2].

The aims of our study were (1) to determine the incidence of GVFL two decades after the start of the Rotterdam Study and (2) to compare risk factor profiles in various OAG outcomes: GVFL, GON, GVFL and GON, GVFL without GON, and GON without GVFL. With this approach we aim to address the question whether OAG with dominating GVFL or dominating GON are different entities or not.

## Methods

### Study population

The Rotterdam Study is a population-based study executed in Ommoord, a district of Rotterdam, the Netherlands. The design and background have been published previously [3]. The research described in this paper is based on Rotterdam Study I (RS-I), which is the original cohort that started in 1990. RS-I included 7983 participants aged 55 years and older. The ophthalmic part of the RS-I started in 1991 and comprised 6806 participants [4]. Follow-up rounds were completed from 1993 to 1995 (RS-I-2; no glaucoma assessments), 1997 to 1999 (RS-I-3) [2], 2002 to 2004 (RS-I-4) [1], and 2009 to 2011 (RS-I-5). Ophthalmic baseline and follow-up examinations included visual field testing, ONH assessment, and measurements of the intraocular pressure and refractive error. The Rotterdam Study was approved by the Medical Ethics Committee of the Erasmus MC and by the Ministry of Health, Welfare and Sport of the Netherlands, implementing the “Wet Bevolkingsonderzoek: ERGO (Population Studies Act: Rotterdam Study)”. All participants provided written informed consent in accordance with the Declaration of Helsinki to participate in the study and to obtain information from their treating physicians.

### Visual field testing and definition of glaucomatous visual field loss

All participants underwent visual field testing using the Humphrey Field Analyzer (HFA; Carl Zeiss Meditec, Jena, Germany). Details have been published before. In short, the visual field of both eyes from each participant was screened with a 52-point supra-threshold test, which tests the 52 points from the Glaucoma Hemifield Test. If a participant did not respond to a light stimulus (6 dB above a threshold-related estimate of the hill of vision) in three or more contiguous points, or four when the defect contained the blind spot, a second supra-threshold test was performed. If the second supra-threshold test showed at least partially (one or more test locations) overlapping abnormalities in the same Hemifield, Goldmann kinetic perimetry (RS-I-1 and RS-I-3; Haag Streit) or full-threshold HFA (RS-I-4, RS-I-5) was performed on both eyes. The Goldmann visual fields were classified according to definitions published before [5]. The full-threshold HFA tests were classified as abnormal if at least one of three criteria was met: (1) a Glaucoma Hemifield Test ‘outside normal limits’, (2) a minimum of three contiguous points in the pattern deviation probability plot with a sensitivity decreased to *p* < 0.05 of which at least one point to *p* < 0.01, or (3) a Pattern Standard Deviation *p* < 0.05. Visual field loss was considered to be present if it was consecutive and reproducible, that is, the abnormalities had to be present on the Goldmann or full-threshold test and on both supra-threshold tests. Defects had to be in the same hemifield and at least one depressed test point had to have exactly the same location on all fields. Fields had to be reliable, that is, false positives and false negatives had to be <33% and fixation losses <20%. Fundus photographs, ophthalmic examination reports, medical histories, and MRI scans of the brain were checked for disorders that could explain the visual field loss. If no other cause could be identified, and no homonymous defects and artifacts like rim artifacts were found, the visual field loss was considered GVFL. Discrepancies were resolved by consensus. If the GVFL was already detected in regular care, additional information was retrieved from the involved ophthalmologist in order to exclude angle-closure and secondary glaucoma. Newly detected cases were invited for a detailed ophthalmic examination. The current study only included GVFL due to OAG, including primary OAG, pseudoexfoliation glaucoma, and pigment dispersion glaucoma.

The pattern of visual field loss was classified using the Ocular Hypertension Treatment Study classification [6]. This classification describes 17 categories, including altitudinal defect, (partial) arcuate scotoma, and a nasal step. A recent prototypical (archetypal) analysis showed that these categories fit well into models of retinal structures (RNFL trajectories) [7]. Hemifield asymmetry was determined by comparing, in the full-threshold HFA tests, the number of abnormal test locations at *p* < 0.5% (black squares) in the total deviation probability plot between the superior and the inferior hemifield.

### Optic nerve head assessment and definition of glaucomatous optic neuropathy

During baseline and the first follow-up with glaucoma assessment (RS-I-3), simultaneous stereo color photos of the ONH were taken at a fixed angle of 20 degrees and analyzed with a computerized image analyzer (Topcon ImageNet System; ImageNet, Topcon Corporation, Tokyo, Japan). For ImageNet, GON was based on the 97.5th percentile of the vertical cup-disc ratio (VCDR). GON was present if VCDR exceeded 0.69 for small discs (up to 2 mm^2^), 0.72 for discs 2.0–2.7 mm^2^, and 0.76 for large discs (>2.7 mm^2^) [1]. During the second and third follow-up rounds (RS-I-4 and RS-I-5, respectively), the Heidelberg Retina Tomograph (HRT; Heidelberg Engineering, Dossenheim, Germany) was used to assess the ONH. The GON cut-off values for HRT were based on the 97.5th percentile of the linear cup-disc ratio (LCDR) and defined as follows: 0.67 for small discs (up to 1.5 mm^2^), 0.71 for discs 1.5–2.0 mm^2^, and 0.76 for large discs (>2.0 mm^2^) [8].

### Definitions of OAG

Participants without GVFL at baseline who developed GVFL in at least one eye during follow-up were considered incident GVFL (iGVFL) cases. Definite OAG was defined as iGVFL with GON [1]. The presence of GON was recorded at the last follow-up examination with both reliable ONH imaging and visual field testing in participants without iGVFL, and at the visit where the iGVFL occurred in participants with iGVFL. Because of the change in ONH assessment technique during the follow-up, we did not study incident GON separately.

### Intraocular pressure and refraction

IOP was measured with Goldmann applanation tonometry (Haag-Streit, Bern, Switzerland). For each eye, the median of three measurements was taken. Refraction was measured with the RM-A2000 autorefractor (Topcon, Tokyo, Japan).

### Statistical analysis

#### Incidence of glaucomatous visual field loss and definite open-angle glaucoma

For each participant, we calculated the time between the baseline visit (RS-I-1) and the last follow-up visit. For cases with iGVFL, the last follow-up visit was the first visit with GVFL. For controls, the last follow-up visit was the last visit with reliable visual field testing. Participants with GVFL at baseline were excluded, as well as participants with no reliable visual field testing at baseline or follow-up.

We calculated the incidence rate (IR) and used the IR to calculate the overall incidence during the entire follow-up. The IR is calculated as the number of cases with iGVFL divided by the number of person years (the sum of follow-up time of all participants). The overall incidence during the entire follow-up was calculated using the formula $1 - e^{{ - \langle {\text{T}}\rangle *{\text{IR}}}} ,$ where *e* is the base of the natural logarithm, 〈T〉 the mean follow-up of all participants, and IR the incidence rate [9]. The incidence rate and overall incidence during the entire follow-up of definite OAG was calculated similarly, based on iGVFL cases with GON (see above). We further calculated the IR of iGVFL in 10-years age categories. For this analysis we used a dynamic population, i.e., participants could contribute person years to subsequent age categories [10]. In this analysis we also stratified for gender.

#### Risk factor analysis and clinical manifestations of OAG

The following baseline risk factors were analyzed: age, gender, IOP, IOP treatment, family history for glaucoma, myopia, diastolic blood pressure (DBP), and body-mass index (BMI). For IOP, we took the highest value of the medians of both eyes (see above) at baseline. IOP treatment was defined as IOP lowering surgery or laser treatment before baseline or the use of IOP lowering medication at baseline. Medication use was based on a fully automated pharmacy database recording including the ATC code (S01E for IOP lowering medication). Surgery and laser treatment were based on interview data with the participant. Family history was considered positive if the participant reported glaucoma in parents, siblings, or offspring during the interview. Spherical equivalent refraction (SE) was calculated as the spherical refractive error plus half of the cylinder. It was stratified in three categories: high myopia, defined as a SE of −4 D or more myopic; low myopia, defined as a SE between −3.99 and −0.01 D; and no myopia, defined as a SE of 0 D and above. For SE, we used the eye with GVFL in case of unilateral GVFL, and a random eye in case of bilateral GVFL and participants without GVFL. The assessment of DBP has been described before [11]. BMI was calculated as mass (in kilograms) divided by the square of height (in meters). Height and weight were measured with indoor clothing and no shoes. In case of missing values for the risk factors, we imputed the missing value to the mean since missing values were present in less than 5% of the participants. In case of cataract extraction in both eyes before baseline, the SE was imputed to the mean; in case of cataract extraction in one eye, the SE of the other eye was taken.

Risk factor analyses were performed using Cox proportional hazards models, with five different outcome measures: (1) iGVFL, (2) GON, (3) iGVFL and GON (definite OAG), (4) iGVFL without GON, and (5) GON without iGVFL. For each analysis, controls were participants without iGVFL and without GON. For this analysis, the last follow-up round with both reliable visual field testing and ONH data was used. Similar to iGVFL (see above, Definitions of OAG), GON was defined as the presence of GON in at least one eye. A Bonferroni-corrected *p* value of 0.01 (0.05/5 analyses) was considered as statistically significant.

In a final comparison, a one-way ANOVA was conducted to compare the mean IOP between participants with GVFL and GON (definitive OAG), GVFL without GON, GON without GVFL, and controls; since there was no homogeneity of variance, the Games-Howell post hoc test was used to compare all groups to each other. A *p* value of 0.05 was considered as statistically significant. All analyses were performed using IBM SPSS Statistics Release 20.0.0 (IBM Corp., Armonk, NY).

## Results

**Fig. 1:**
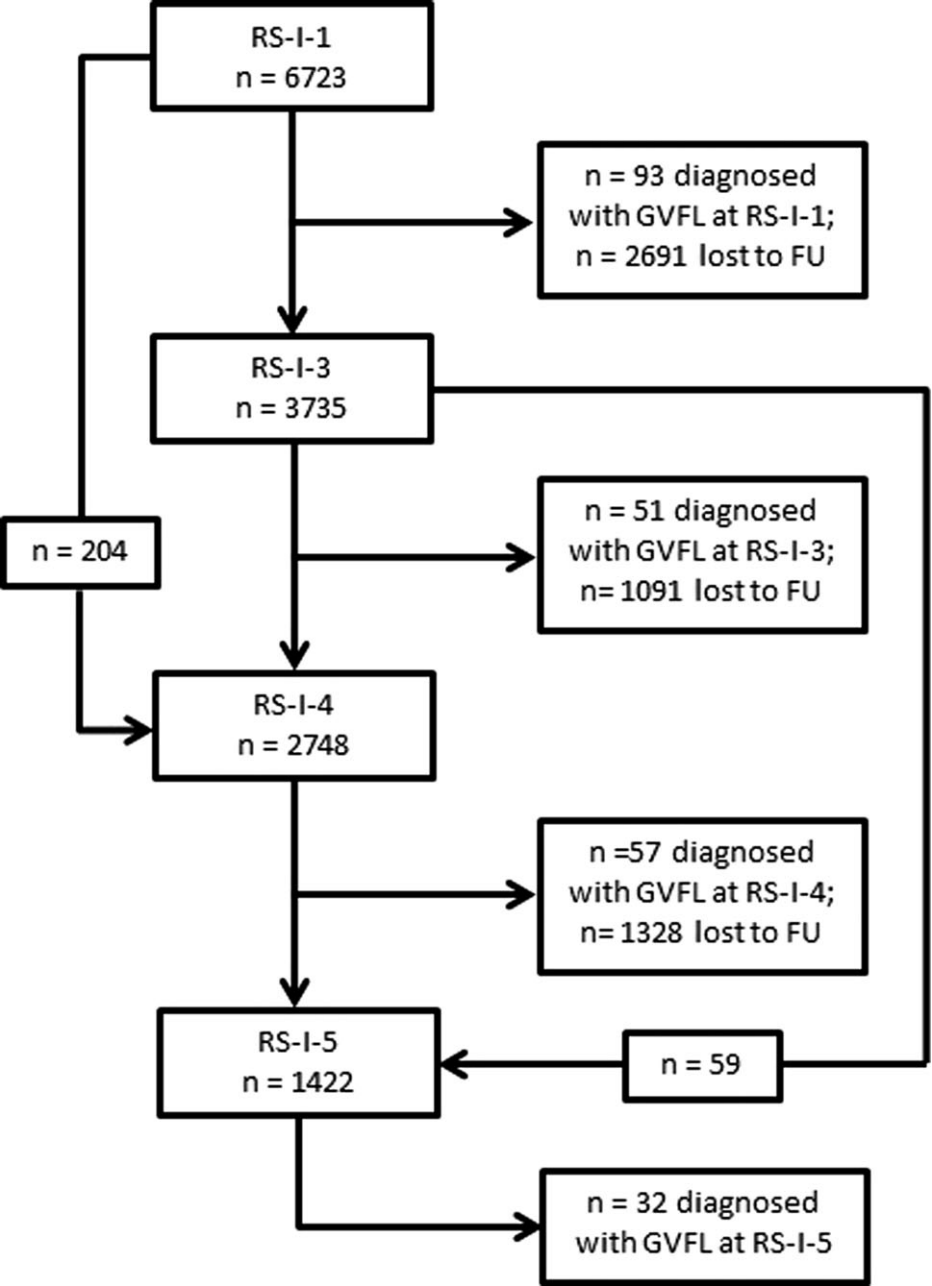
Flow diagram which shows the number of participants with reliable visual field testing in the different follow-up rounds. *FU* follow-up, *GVFL* glaucomatous visual field loss, *RS-I* Rotterdam Study I, *RS-I-1* baseline examinations, *RS-I-3* first follow-up round, *RS-I-4* second follow-up round, *RS-I-5* third follow-up round

After exclusion of participants with GVFL at baseline and participants without visual field testing at follow-up, 3939 participants were eligible for the study (see Fig. 1). Table 1 presents the baseline characteristics with univariable comparisons. Of the 3939 participants, 140 developed GVFL during one of the follow-up rounds. The median follow-up was 11.1 (IQR 6.8–17.2; range 5.0–20.3) years, the mean follow-up 12.1 years, and the total follow-up 47,710 person-years. The incidence rate was 2.9 (95% CI 2.4–3.4) per 1000 person years; the 12-years incidence was 3.5 (2.9–4.0)%. For definite OAG, the incidence rate per 1000 person-years and 12-years incidence were 1.0 (0.7–1.3) and 1.2 (0.9–1.5)%, respectively. Table 2 presents age- and gender-specific incidence rates of GVFL. The incidence rate increased from 0.8 at age 55–64 years to 12.7 per 1000 person years at age 85 and above.

**Table 1:** Baseline demographic and clinical characteristics of participants with and without iGVFL, presented as mean ± SD or percentages

	No GVFL (n = 3799)	iGVFL (n = 140)	*p* value
Age (years)	65.7 ± 6.8	67.2 ± 7.0	0.01
Gender (female)	58.6%	54.3%	0.32
IOP (mmHg)	15.0 ± 3.1	17.0 ± 4.4	<0.001
IOP Rx	1.6%	10.0%	<0.001
FH	8.0%	17.9%	<0.001
Myopia			0.56
Low myopia	20.7%	19.3%	
High myopia	5.0%	7.1%	
DBP (mmHg)	73.6 ± 10.8	72.8 ± 12.0	0.36
BMI (kg/m^2^)	26.3 ± 3.5	25.7 ± 3.1	0.03

*BMI* body-mass index, *DBP* diastolic blood pressure, *FH* positive family history for glaucoma, *iGVFL* incident glaucomatous visual field loss, *IOP* intraocular pressure, *IOP Rx* intraocular pressure lowering treatment

**Table 2:** Incidence rates of glaucomatous visual field loss as a function of age and gender

Age group (years)	Number of cases	Person years at risk	IR per 1000 person years (95% CI)	12-years risk (95% CI)
Male				
55–64	2	3950	0.5 (0.0–1.2)	0.6 (−0.2–1.4)%
65–74	23	10,180	2.3 (1.3–3.2)	2.7 (1.6–3.7)%
75–84	32	4951	6.5 (4.2–8.7)	7.5 (4.9–9.9)%
85+	7	478	14.6 (3.8–25.5)	16.1 (4.5–26.3)%
Overall	64	19,560	3.3 (2.5–4.1)	3.9 (2.9–4.8)%
Female				
55–64	5	5331	0.9 (0.1–1.8)	1.1 (0.1–2.1)%
65–74	17	13,615	1.2 (0.7–1.8)	1.5 (0.8–2.2)%
75–84	40	8030	5.0 (3.4–6.5)	5.8 (4.0–7.5)%
85+	14	1174	11.9 (5.7–18.2)	13.3 (6.6–19.6)%
Overall	76	28,150	2.7 (2.1–3.3)	3.2 (2.5–3.9)%
Total				
55–64	7	9281	0.8 (0.2–1.3)	0.9 (0.2–1.6)%
65–74	40	23,795	1.7 (1.2–2.2)	2.0 (1.4–2.6)%
75–84	72	12,982	5.5 (4.3–6.8)	6.4 (5.0–7.9)%
85+	21	1652	12.7 (7.3–18.2)	14.1 (8.4–19.6)%
Overall	140	47,710	2.9 (2.4–3.4)	3.5 (2.9–4.0)%

*CI* confidence interval, *IR* incidence rate

Of the 140 iGVFL cases, 27 (19.3%) had bilateral iGVFL at the time of diagnosis, 52 (37.1%) had iGVFL in only the right eye, and 61 (43.6%) had iGVFL in only the left eye (*p* = 0.42). Of these 113 unilateral cases, 8 cases developed GVFL in the second eye during a later follow-up round. Of all the iGVFL cases, 89 were diagnosed with the full threshold HFA (RS-I-4 and RS-I-5) of which 17 had bilateral iGVFL. Seven of these 106 (89 + 17) eyes (six cases) showed an altitudinal defect (all in the upper hemifield); 30 eyes showed an arcuate scotoma in one hemifield (20 upper hemifield, 10 lower hemifield), and 50 eyes showed an arcuate scotoma in both hemifields. Fifteen eyes showed a partial arcuate scotoma (8 upper hemifield, 4 lower hemifield, and 3 in both hemifields); 4 eyes showed a nasal step (1 upper hemifield, 2 lower hemifield, 1 both hemifields). Overall, 36 eyes had a scotoma in the superior hemifield only, 16 in the inferior hemifield only (*p* = 0.01), and 54 in both hemifields. In 56 of the 106 eyes (52.8%) the upper hemifield was more severely affected than the lower hemifield (not significantly different from 50%; *p* = 0.35). This indicates that, if scotomata are present in both hemifields, the loss is more pronounced inferiorly than superiorly. Indeed, in the 54 eyes with a scotoma in both hemifields, on average 11.9 test locations were affected in the upper hemifield and 14.3 in the inferior hemifield (at *p* < 0.5% (black squares) in the total deviation probability plot).

**Fig. 2:**
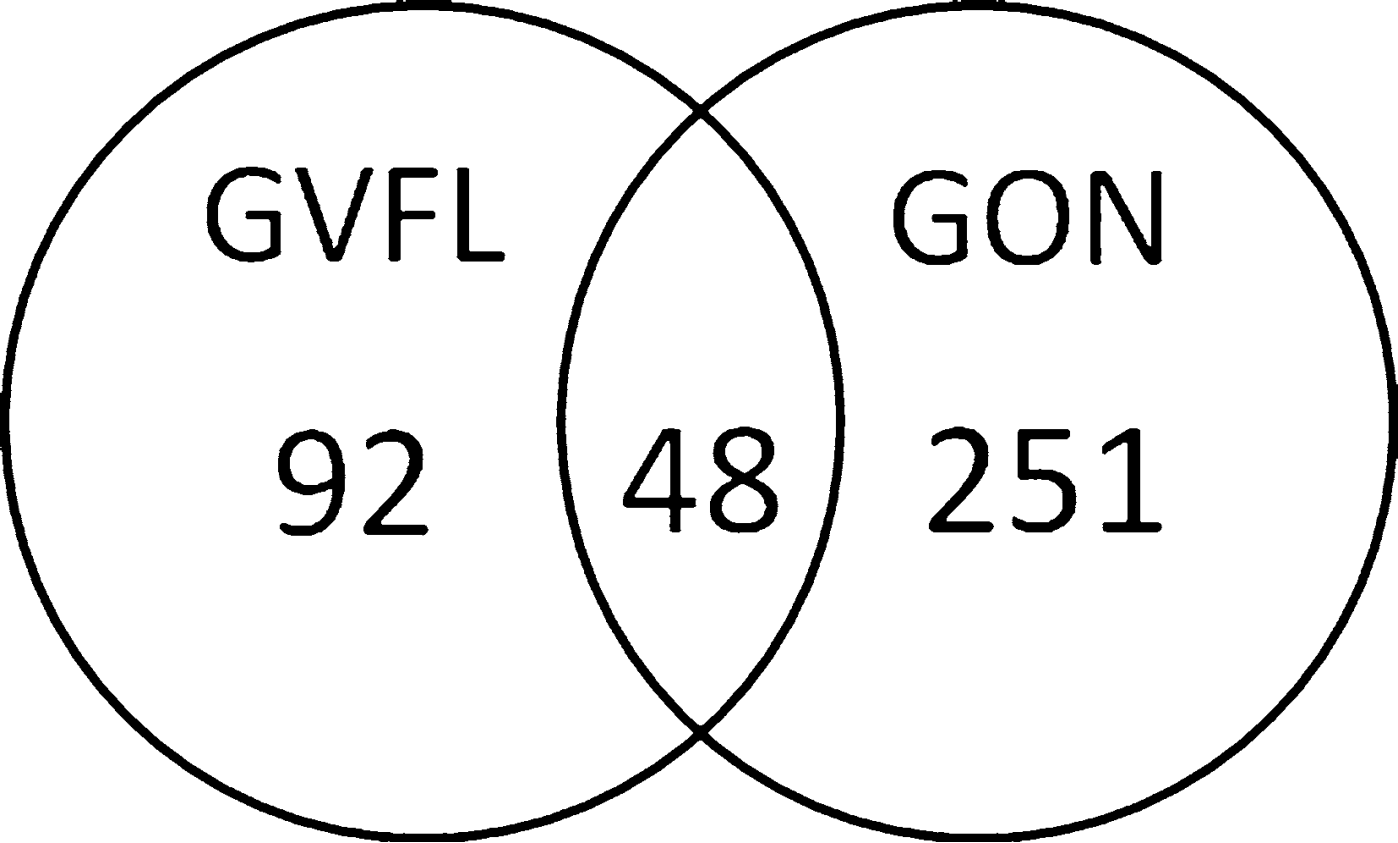
Number of participants with incident glaucomatous visual field loss (iGVFL), glaucomatous optic neuropathy (GON), or both. The presence of GON was recorded at the last follow-up examination with both reliable ONH imaging and visual field testing in participants without iGVFL, and at the visit where the iGVFL occurred in participants with iGVFL

**Fig. 3:**
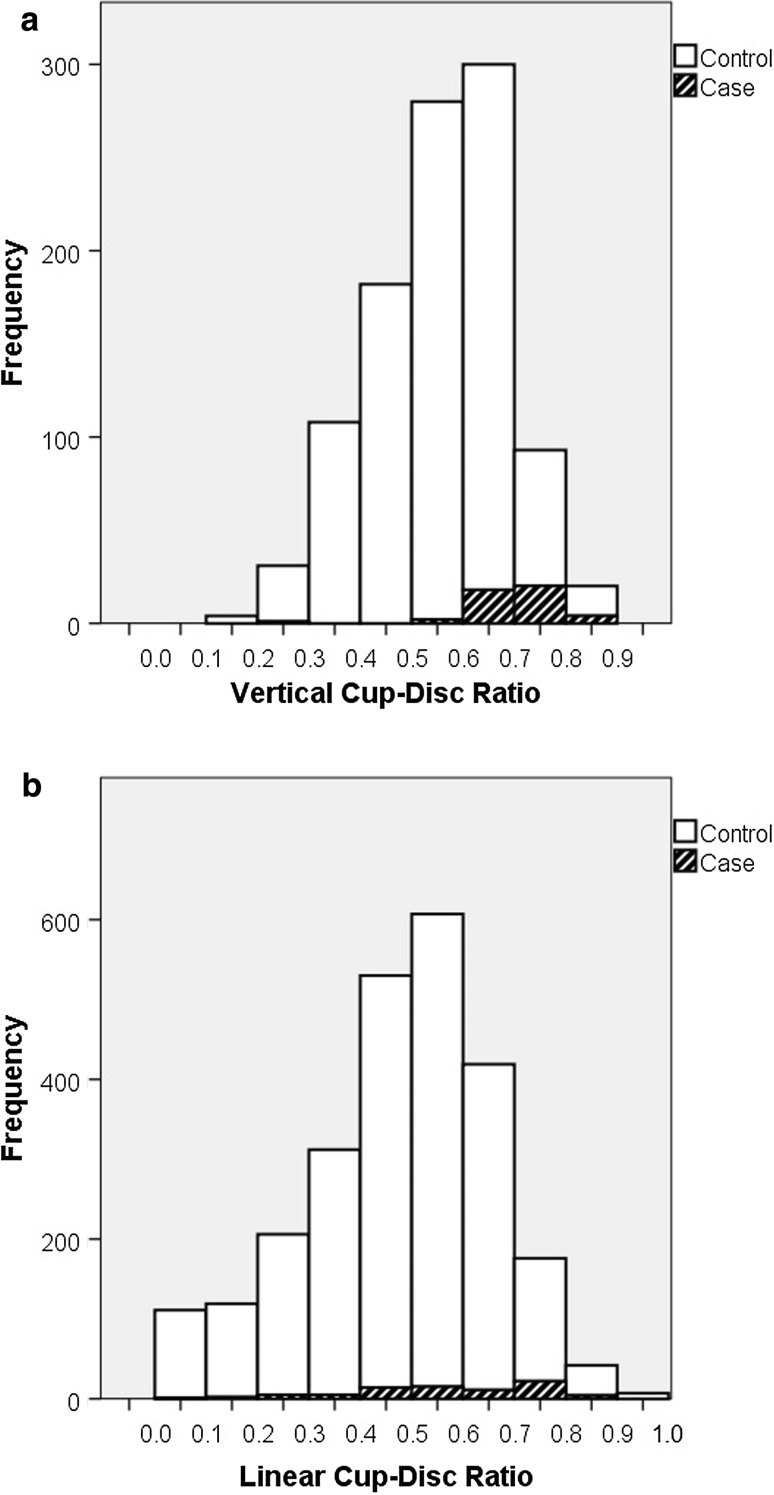
Distribution of vertical cup-disc ratio (**a**) and linear cup-disc ratio (**b**) in cases with incident glaucomatous visual field loss (iGVFL; *in black pattern*) and controls without GVFL (*in white*)

Of the 140 cases with iGVFL, 24 participants had GON at baseline (as assessed with ImageNet) and 48 had GON at follow-up (as assessed with HRT). Another 251 participants had GON at follow-up but no iGVFL (Fig. 2). Of the participants without GON at the time that iGVFL was diagnosed, 10 developed GON in a next follow-up round. Figure 3 shows the VCDR (A; RS-I-3) and LCDR (B; RS-I-4 and RS-I-5) distributions of cases with iGVFL and controls. Although two-third of the cases with iGVFL did not have GON formally, the distributions of the iGVFL cases were clearly shifted towards higher VCDR/LCDR values, when compared to the controls (participants without iGVFL).

The differences in risk factors between participants with iGVFL and/or GON are shown in Table 3. IOP and age were significant risk factors for all outcomes. A positive family history was associated with iGVFL, GON, and definite OAG. Gender, myopia, and DBP were not significantly associated with any of the outcomes; BMI appeared to be associated with various outcomes, but only for GON at the Bonferroni-corrected *p* = 0.01.

**Table 3:** Results of cox-regression models with different outcomes of glaucoma (see Fig. 2; in all models, controls were participants without iGVFL and without GON [n = 3548])

	iGVFL (n = 140)		GON (n = 299)		iGVFL and GON (n = 48)		iGVFL without GON (n = 92)		GON without iGVFL (n = 251)	
	Hazard ratio (95% CI)	*p*	Hazard ratio (95% CI)	*p*	Hazard ratio (95% CI)	*p*	Hazard ratio (95% CI)	*p*	Hazard ratio (95% CI)	*p*
Age (years)	1.09 (1.06–1.12)	<0.001	1.11 (1.10–1.13)	<0.001	1.09 (1.04–1.14)	<0.001	1.09 (1.06–1.13)	<0.001	1.12 (1.10–1.14)	<0.001
Gender (f)	0.78 (0.56–1.10)	0.16	0.96 (0.76–1.21)	0.72	0.89 (0.49–1.60)	0.69	0.74 (0.49–1.12)	0.15	0.97 (0.75–1.25)	0.80
IOP (mmHg)	1.14 (1.10–1.19)	<0.001	1.11 (1.08–1.15)	<0.001	1.18 (1.11–1.24)	<0.001	1.13 (1.06–1.20)	<0.001	1.09 (1.05–1.13)	<0.001
IOP Rx	2.61 (1.35–5.04)	<0.01	1.57 (0.92–2.71)	0.10	3.00 (1.07–8.44)	0.04	2.43 (1.00–5.92)	0.05	1.11 (0.54–2.30)	0.77
FH	2.15 (1.37–3.38)	<0.001	1.67 (1.17–2.37)	<0.01	2.85 (1.40–5.79)	<0.01	1.85 (1.01–3.38)	0.05	1.49 (0.99–2.24)	0.06
Low myopia	0.97 (0.64–1.49)	0.90	0.98 (0.73–1.32)	0.89	1.24 (0.62–2.47)	0.55	0.83 (0.48–1.44)	0.51	0.93 (0.67–1.30)	0.67
High myopia	1.53 (0.80–2.95)	0.20	1.46 (0.93–2.29)	0.10	1.45 (0.44–4.75)	0.54	1.54 (0.71–3.38)	0.28	1.47 (0.90–2.39)	0.12
DBP	1.00 (0.98–1.01)	0.81	1.00 (0.99–1.01)	0.58	0.99 (0.96–1.01)	0.30	1.00 (0.99–1.02)	0.65	1.01 (0.99–1.02)	0.27
BMI	0.94 (0.89–0.99)	0.02	0.96 (0.93–0.99)	0.01	0.92 (0.84–1.01)	0.09	0.94 (0.88–1.00)	0.07	0.96 (0.93–1.00)	0.05

*95% CI* 95% confidence interval, *BMI* body-mass index, *DBP* diastolic blood pressure, *FH* positive family history for glaucoma, *GON* glaucomatous optic neuropathy, *iGVFL* incident glaucomatous visual field loss, *IOP* intraocular pressure, *IOP Rx* intraocular pressure lowering treatment

The mean baseline IOP in participants with definite OAG was 18.4, in iGVFL without GON 16.3, in GON without iGVFL 15.8, and in the controls 15.0 mmHg. Post hoc comparisons using the Games-Howell test indicated that the mean IOP was significantly different between the controls and all other groups. Furthermore, the mean IOP was significantly different between definite OAG and participants with GON without iGVFL (*p* = 0.014). There was no significant difference in IOP between definite OAG and iGVFL without GON (*p* = 0.09) and iGVFL without GON and GON without iGVFL (*p* = 0.67).

## Discussion

In this study, the 12-years incidences of GVFL and definite OAG were 3.5 and 1.2%, respectively, and the corresponding incidence rates 2.9 and 1.0 per 1000 person years. The 12-years incidence of GVFL increased from 0.8 to 12.7% in the age range studied. Unilateral GVFL occurred as often in the right eye as in the left eye; if only one hemifield was affected, GVFL was more often present in the superior hemifield than in the inferior hemifield. However, the majority of eyes had GVFL in both hemifields and, overall, the glaucomatous damage did not differ between the hemifields. About one-third of the cases with iGVFL had GON. Our data do not support the hypothesis that OAG with dominating GVFL or dominating GON are different entities, as depicted by the finding that the various clinical manifestations of OAG did not differ noticeably in their associations with the established OAG risk factors studied.

The incidence rate of 2.9 per 1000 person years was similar to the incidence rate that was found previously in the Rotterdam Study after 10 years of follow-up [1]. In a population-based study in Italy, Cedrone et al. [12] found a 12-years incidence of OAG of 3.8% (95% CI 2.3–6.2), quite similar to our 3.5%. Their definition of OAG was GVFL plus IOP ≥ 21 mmHg or VCDR ≥ 0.5 or VCDR asymmetry ≥ 0.2. Hence, their incidence of GVFL without other criteria would probably be higher. On the other hand, they only performed visual field testing in suspect glaucoma (IOP ≥ 21 mmHg or VCDR ≥ 0.5 or VCDR asymmetry ≥ 0.2) and at random in 50% of the other individuals. In this way they will have missed some iGVFL cases, being the cases without elevated IOP and without a clearly excavated ONH.

In the study from Cedrone et al. [12], 53% of the incident OAG cases had unilateral visual field loss. Data concerning the occurrence in right or left eyes was not provided. A ratio of 1:1 for uni- and bilateral OAG was also found in the Blue Mountains Eye Study [13]. We found a greater percentage (81%) of unilateral cases than these studies. This difference could be explained by the fact that we examined our individuals on regular time intervals and thus detected the GVFL in an earlier stage of the disease. The time between the two examinations in the study from Cedrone et al. was 12 years, while the Blue Mountains Eye Study described also prevalent cases. In the Blue Mountains Eye Study, 34.2% of 152 eyes with GVFL had defects in only the upper hemifield, 40.1% in only the lower hemifield (*p* = 0.13), and 25.7% had defects in both hemifields. This absence of a clear hemifield preference agrees with our study: we found—in eyes with only one affected hemifield—a predominance of superiorly over inferiorly located scotomata, but if both hemifields were affected, the loss was predominantly located inferiorly. The clinical implication of this finding is that patients with glaucomatous loss in the inferior hemifield are more likely to develop loss in the intact hemifield, compared to patients with glaucomatous loss in the superior hemifield.

It has been postulated that NTG differs from HTG in optic nerve head appearance. Caprioli et al. [14] found thinner optic disc rim in NTG patients (n = 34) compared to HTG patients (n = 41), especially in the inferior and inferotemporal area. Iester et al. [15] compared HRT parameters between HTG patients (n = 132) and NTG patients (n = 50) and found no statistically significant differences in any of the parameters. This is in line with the results of our unbiased study (as argued in the Introduction section, NTG might bias towards more pronounced ONH abnormalities in a clinical setting). We were not able to locate studies addressing asymmetry in left/right eye and/or inferior/superior hemifield occurrence of GVFL.

We found no association between myopia and any of the OAG outcomes. A meta-analysis showed that myopia was associated with glaucoma (odds ratio of 1.9) [16]. Previously, we also found an association between high myopia and OAG in the Rotterdam Study (HR 2.3 [1.2–4.5], *p* = 0.01) [1]. However, of the 32 participants who developed GVFL during the latest follow-up round only one had high myopia and therefore the effect of the association disappeared. Our finding suggests that (high) myopia may mainly play a role in the development of OAG at a younger age. After all, the mean age of the participants at the latest follow-up round (RS-I-5) was 79.5 years (to be compared to 66 years at baseline). This is supported by a recent study in which participants with high myopia developed OAG earlier than others [17]. A similar phenomenon occurred for gender. We previously identified male gender as a risk factor for glaucoma (HR 1.62 [1.10–2.38], *p* = 0.015) [1]. The current study found a higher IR among males but yielded no significant associations for gender in the risk factor analysis, apparently related to an excess of females (27) amongst the 32 most recently diagnosed iGVFL cases (72%). This suggests that males tend to develop OAG at an earlier age. However, the wide confidence intervals in the individual age and gender categories do not permit firm conclusions.

We found a nominally significant association between BMI and GVFL (*p* = 0.02) and BMI and GON (*p* = 0.01), which were not significant after correction for multiple testing. The associations between BMI and the other outcomes were not significant. However, the hazard ratios were all in the same—protective—direction (0.92–0.96). Other studies also found a protective effect of BMI on OAG [18–22]. Furthermore, previous studies found that a higher BMI was associated with small cup-disc ratios or cup areas [23–25], which is in line with our finding that a higher BMI is protective for GON.

In our study, DBP was not associated with OAG. A recent meta-analysis, which included 27 studies that investigated the relationship between blood pressure and glaucoma, found a pooled relative risk of 1.16 (95% CI 1.05–1.28) for the effect of hypertension (not separately studied for systolic blood pressure and DBP) on OAG [26]. However, they showed some heterogeneity across studies (I^2^ 34.5%; 18 studies reported a positive association and 9 studies reported an inverse or no association) and the effect was only significant in cross-sectional studies; the pooled relative risk of two longitudinal studies was 1.05 (0.69–1.59). Clearly, the power was limited here, but—generally speaking —longitudinal studies are more informative concerning a causal relationship between a risk factor and a disease. Our results, together with the previous results from the two longitudinal studies, suggest that there is no clear association between blood pressure and OAG. Studying blood pressure as a linear variable implies the risk of overlooking non-linear associations, for example an increased risk for those with a very low or a very high blood pressure. In our study, entering DBP in quartiles did not reveal any association either (data not shown), suggesting the absence of a clear nonlinear relationship between OAG and DBP.

A strong point of our study is that all participants underwent visual field testing, regardless of ONH abnormalities or IOP measurements. We showed that two-third of the iGVFL cases had no ONH abnormalities exceeding the 97.5th percentile. Studies who performed only visual field testing in subjects with suspicious ONH findings may thus miss many OAG cases. A limitation of the study is the relatively low number of cases, which is inherent to the low incidence of OAG in the general population but hampers detailed risk factor analyses.

In conclusion, we found a 12-years incidence of 3.5% for GVFL. Risk factor profiles were similar for the different clinical manifestations of OAG. We confirmed the associations between OAG and age, IOP, and family history. We found no association for either gender or myopia, and hypothesized that these factors may particularly be related to OAG with a younger age of onset.
